# Dealloying of gold–copper alloy nanowires: From hillocks to ring-shaped nanopores

**DOI:** 10.3762/bjnano.7.127

**Published:** 2016-09-29

**Authors:** Adrien Chauvin, Cyril Delacôte, Mohammed Boujtita, Benoit Angleraud, Junjun Ding, Chang-Hwan Choi, Pierre-Yves Tessier, Abdel-Aziz El Mel

**Affiliations:** 1 Institut des Matériaux Jean Rouxel, IMN, Université de Nantes, CNRS, 2 rue de la Houssinière B.P. 32229, 44322 Nantes cedex 3, France; 2 CEISAM, Université de Nantes, CNRS, 2 rue de la Houssinière B.P. 32229, 44322 Nantes cedex 3, France; 3 Department of Mechanical Engineering, Stevens Institute of Technology, Hoboken, NJ 07030, USA

**Keywords:** copper, dealloying, gold, hillocks, nanoporous

## Abstract

We report on a novel fabrication approach of metal nanowires with complex surface. Taking advantage of nodular growth triggered by the presence of surface defects created intentionally on the substrate as well as the high tilt angle between the magnetron source axis and the normal to the substrate, metal nanowires containing hillocks emerging out of the surface can be created. The approach is demonstrated for several metals and alloys including gold, copper, silver, gold–copper and gold–silver. We demonstrate that applying an electrochemical dealloying process to the gold–copper alloy nanowire arrays allows for transforming the hillocks into ring-like shaped nanopores. The resulting porous gold nanowires exhibit a very high roughness and high specific surface making of them a promising candidate for the development of SERS-based sensors.

## Introduction

Improvement in nanoscience involves fundamental evolution in the synthesis and nanofabrication approaches, which allows for producing nanostructures with complex shapes and morphologies not possible to achieve using classical routes [1]. Physical vapor deposition (PVD) is a very simple and efficient process usually used for the growth of thin films finding application in a wide range of fields including microelectronics, optics, biotechnology and protective coatings [2]. In the last few years, PVD started to be used for the growth of one-dimensional (1D) nanostructures such as nanowires [3], nanorods [4] and nanosprings [5]. Although such approaches are very practical, controlling the growth of the material to obtain 1D nanostructures with a hierarchical structuring stays very challenging.

In thin film deposition processes, the surface of the substrate is a key point to control the growth of thin films. Indeed, since the surface of the substrate is the starting point for film growth, the surface topography (roughness, defects, impurities) has a direct impact on the final structure and morphology of the material. As a consequence of the presence of substrate surface defects, the films deposited by a PVD process may contain undesired defects such as hillocks, pinholes, and craters [6–7]. The formation of hillocks has been encountered in case of various processes such as evaporation and sputtering [7–9], ion beam assisted deposition (IBAD) [10–11], chemical vapor deposition (CVD) [12–13] and electroplating [14]. Hillocks are the outcome of a nodular growth taking place because of a non-homogenous surface topography with hills distributed over the surface. During PVD the growth of the material occurs preferredly on the hills acting as nucleation sites [15–16]. The local self-shadowing effect at these nucleation sites is the main driving force promoting the formation of hillocks [17–19]. This nodular growth is characterized by the formation of hillocks emerging from the surface of the films with a conical shaped top originating from the narrow angular distribution of the flux of the species [20].

**Figure 1:**
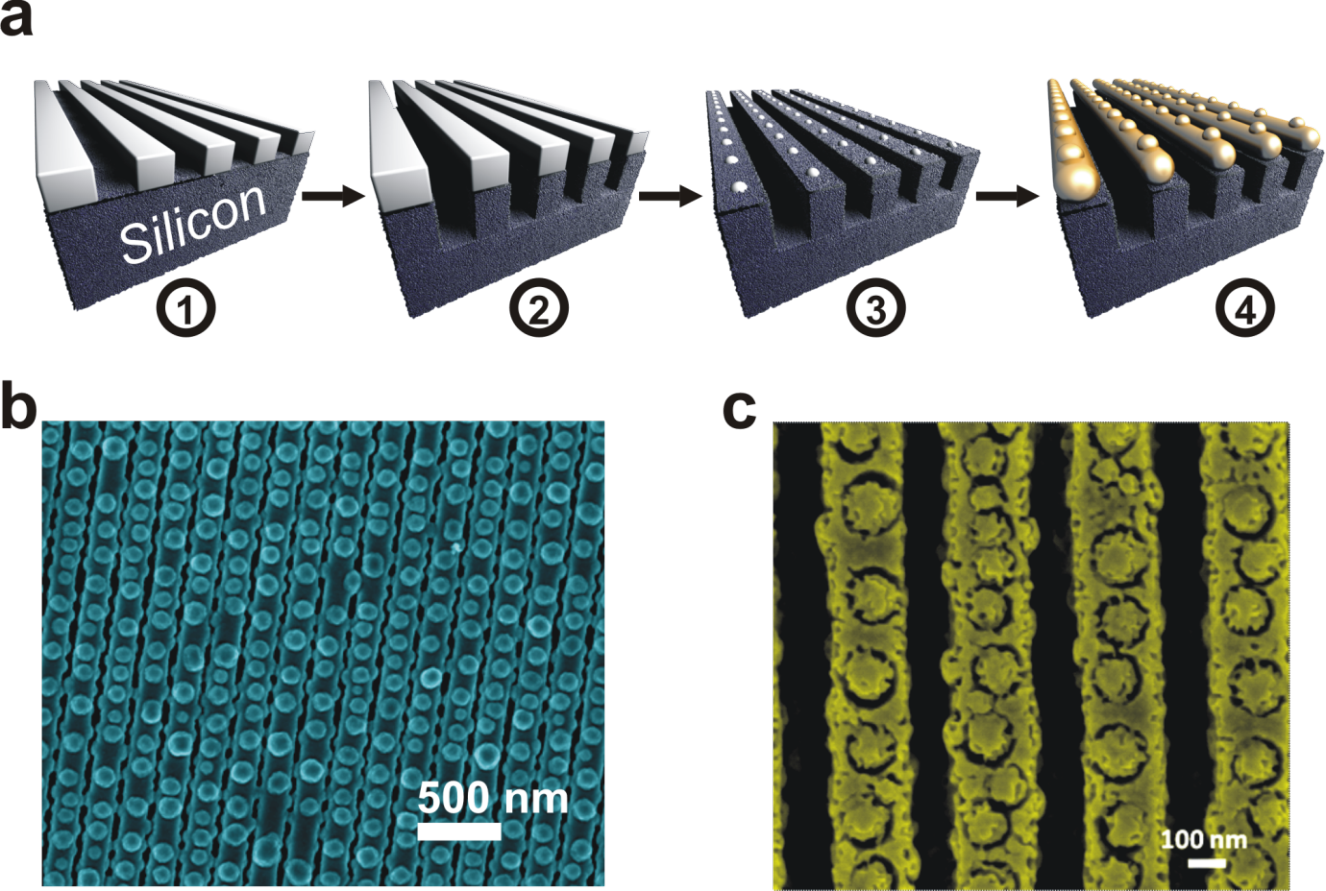
(a) Scheme of the fabrication process developed in this work to prepare metallic nanowire arrays with a nodular morphology. The stages of the process are as follow: (1) nanopatterning on a photoresist film on a silicon substrate using laser interference lithography, (2) etching the silicon substrate through the photoresist mask to form nanograted silicon structures, (3) partial etching of the photoresist using oxygen plasma leaving behind residues of photoresist and (4) deposition of metal over the patterned substrate by magnetron sputtering allowing to grow nanowires with a nodular morphology. SEM micrograph showing the typical morphology of metal nanowires: (b) as grown and (c) after electrochemically dealloying.

In this paper, we report on a strategy to grow hillocks on the surface of metal nanowires (Figure 1a). We show how controlling accurately the growth of such defects can be of real benefit for engineering the surface of nanowires. At first, a photoresist film is deposited by spin coating on a silicon substrate and then patterned using laser interference lithography (Figure 1a); the process is described in details elsewhere [21]. Then, the silicon is etched through the photoresist mask using SF_6_/O_2_ plasma to create nanograted silicon structures (Figure 1a(2)). After this step, an oxygen plasma treatment is applied to partially etch the photoresist lines and transform them into residues (Figure 1a(3)). The existence of photoresist residues is related to the non-homogenous etching of the polymers forming the photoresist. In the last stage, the metal is deposited by magnetron sputtering over the prepared substrate to form an array of nanowires containing hillocks (Figure 1a(4)). We have demonstrated in a previous work that without photoresist residues, such an approach results in nanowires with a smooth surface [22]. The formation of hillocks in the present work originates from the nodular growth taking place because of the photoresist residues acting as localized defects on the surface of the nanograted structures. We demonstrate the ability of forming nanowires with such a special morphology with various metals including gold (Au), copper (Cu), silver (Ag), gold/copper (Au–Cu) and gold/silver (Au–Ag) alloys (Figure 1b). We further show that by applying a dealloying process to Au–Cu alloy nanowires, one can synthesize nanoporous nanowires with a special morphology (Figure 1c) that cannot be obtained when dealloying Au–Cu nanowires with a smooth surface.

## Results and Discussion

**Figure 2:**
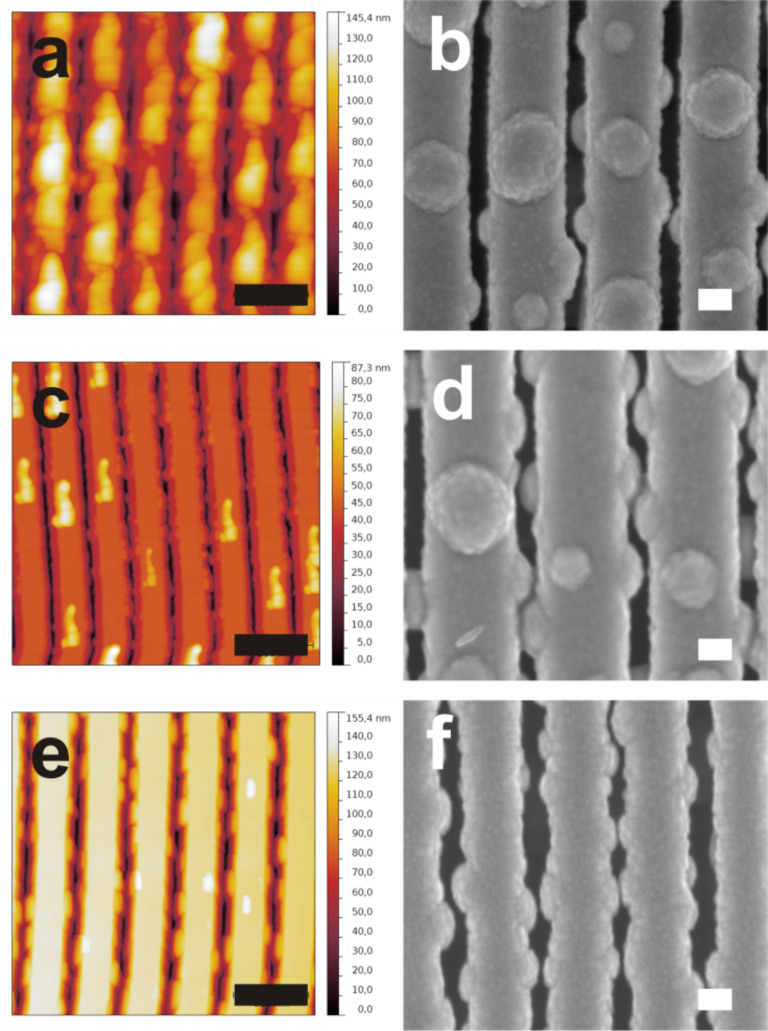
AFM images of substrate and the corresponding SEM micrograph after deposition of 220 nm of metal. Three different types of substrates were used. The defects characteristics vary from a substrate to another: (a,b) large and dense in case of the first type, (c,d) small and dispersed in the second type, (e,f) in third type of substrate the surface defects were completely removed using a piranha solution. The AFM and SEM images are representative of the substrates surface but were not recorded on the same areas. Black scale bar: 500 nm, white scale bar: 100 nm.

There are several factors that influence the nodular growth including the characteristics of the photoresist residues (size and density) as well as the metal deposition conditions. Figure 2 shows the influence of the size and density of defects on the final morphology of metal nanowires (copper in this case). For substrates with large and dense photoresist residues (Figure 2a), the metal nanowires exhibit a very rough morphology with the appearance of a high amount of large hillocks over the surface (Figure 2b). Decreasing the size and density of the photoresist residues (Figure 2c) reduces the size and density of the hillocks (Figure 2d). To confirm that the nodular growth is a consequence of the presence of the photoresist residues, we have carried out a metal deposition on substrates cleaned with piranha solution and without any residues of photoresist (Figure 2e). As expected, the surface of the obtained nanowires is smooth, which is in full agreement with our previous work [22]. We have further performed deposition of different metals (e.g., Au, Cu and Ag) and alloys (e.g., Au–Ag and Au–Cu) on substrates with large amounts of residue. The deposition time was selected in such a way that the average diameter of the nanowires was about 200 nm. The diameters of the hillocks, determined from statistical evaluation of plan-view SEM images of the nanowires (Supporting Information File 1, Figure S1), are almost constant for any metal (about 125 nm). The transmission electron microscopy (TEM) cross-section images of the Au–Cu alloy nanowires show that the hillocks exhibit the same multilayered structure observed for the main body of the nanowire (Supporting Information File 1, Figure S2). This multilayered structure, not present in case of elemental metals, is related to the substrate rotation during the co-sputter deposition [23–24].

**Figure 3:**
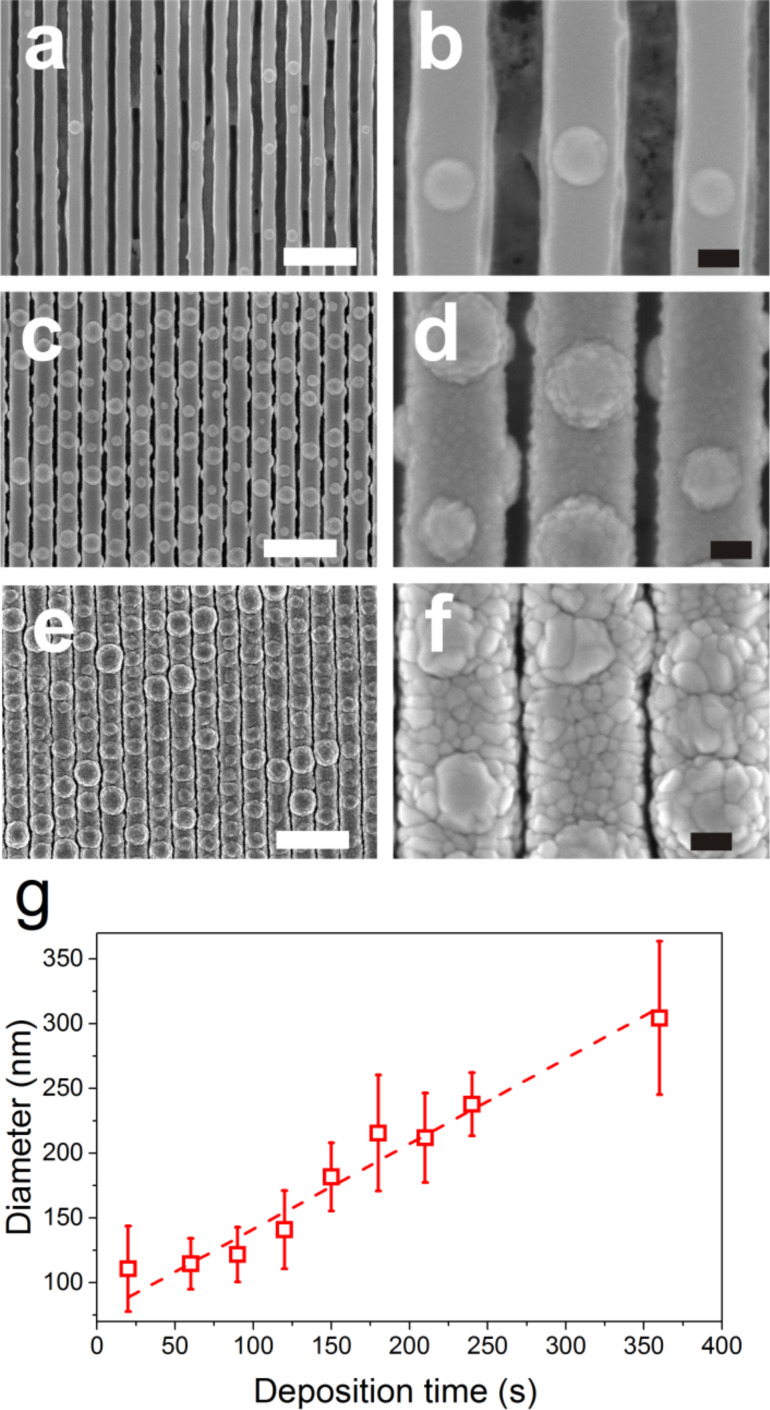
SEM images after (a,b) 30, (c,d) 220 and (e,f) 360 s of deposition time of copper. (g) Evolution of the hillocks diameter as a function of the deposition time. The high magnification SEM images were not recorded on the same areas scanned at low magnification but are representative of the sample surface. White scale bar: 1 µm, black scale bar: 100 nm.

To further study the kinetic of the nodular growth, we have fixed the rotation speed to 5 rpm and carried out metal depositions for various durations (between 20 and 360 s resulting in a thickness between 20 and 360 nm) on nanopatterned substrates with high amounts of photoresist residues. When examining the SEM images, one can remark a clear morphological evolution as a function of the deposition time (Figure 3a–f). After 30 s of deposition, dispersed hillocks with a small size can be seen (Figure 3a and b). When increasing the deposition time to 220 s, many large hillocks can be obtained (Figure 3c and 3d). While an equivalent amount of hillocks was also observed for 360 s of metal deposition (Figure 3e), an increase in hillocks diameter can be noticed (Figure 3f). The diameter of the formed hillocks, extracted from the SEM images, is found to increase linearly from 60 to 300 nm when the increasing deposition time from 20 to 360 s (Figure 3g). Atomic force microscopy (AFM) measurements show no significant evolution in hillocks height as a function of the deposition time (Supporting Information File 1, Figure S3). It remains constant at about 45 nm corresponding to the height of the photoresist residues initially covering the substrate.

**Figure 4:**
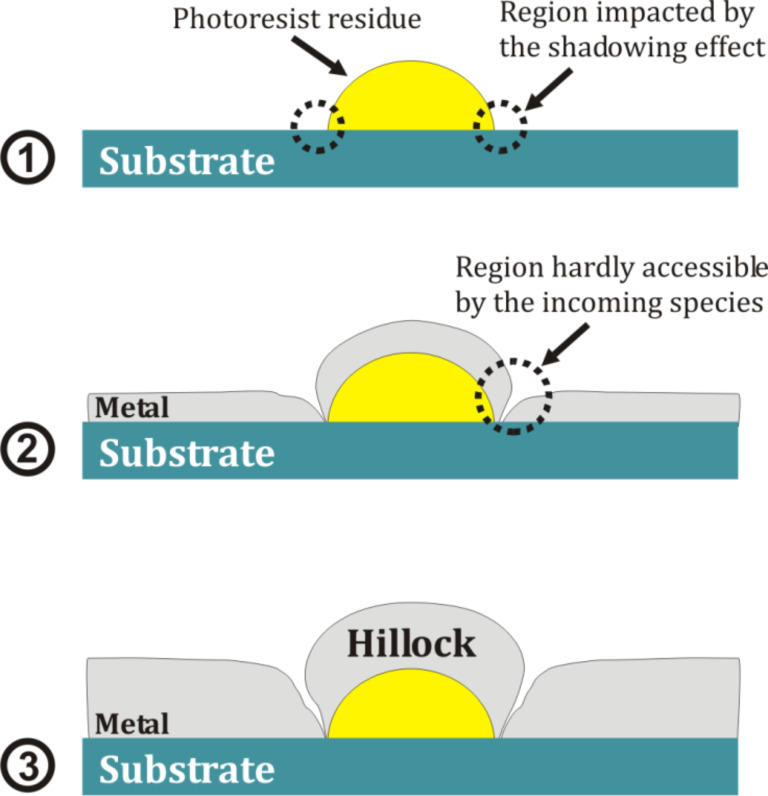
Schematic illustrating the different formation stages of hillocks during the deposition of a metal in the presence of a photoresist residue in a form of nanodome. (1) Substrate before deposition, (2) early and (3) advanced stage of the formation of a metal hillock over the photoresist nanodome as a consequence to the local shadowing effect.

Based on our experimental data presented above, a mechanistic scenario explaining the formation of hillocks can be suggested. In case of a single photoresist residue resting on the substrate surface as illustrated in Figure 4(1), the corner regions at the basis of the nanodome represent the zones impacted by a shadowing effect. This shadowing effect is enhanced by the fact that the angle between the magnetron source axis and the normal to the substrate was 30°. During the early stage of deposition (Figure 4(2)), the metal grows non-uniformly on the photoresist residues. The film is thick at the top region of the photoresist residue and thin at its basis. This is related to the fact that the corner regions between the substrate and the photoresist residue can be hardly reached by the sputtered species because of the shadowing effect from the residue. Since the metal deposition is carried out at a low temperature, the species reaching the surface of the substrate are not capable to diffuse over a long distance and to reach the corner regions. The shadowing effect is amplified when the film grows thicker during the deposition as the size of the residue becomes larger and the substrate regions surrounding the residue also become covered by a metal layer. As a consequence, the shadowing effect results in the formation of open boundaries (Figure 4(3)) between the metal layer covering the photoresist residues and the rest of the film coating the substrate because high points on the growing surface receive more material flux than valleys do, particularly when in addition a significant oblique component is present as it is the case in our process [16,20].

**Figure 5:**
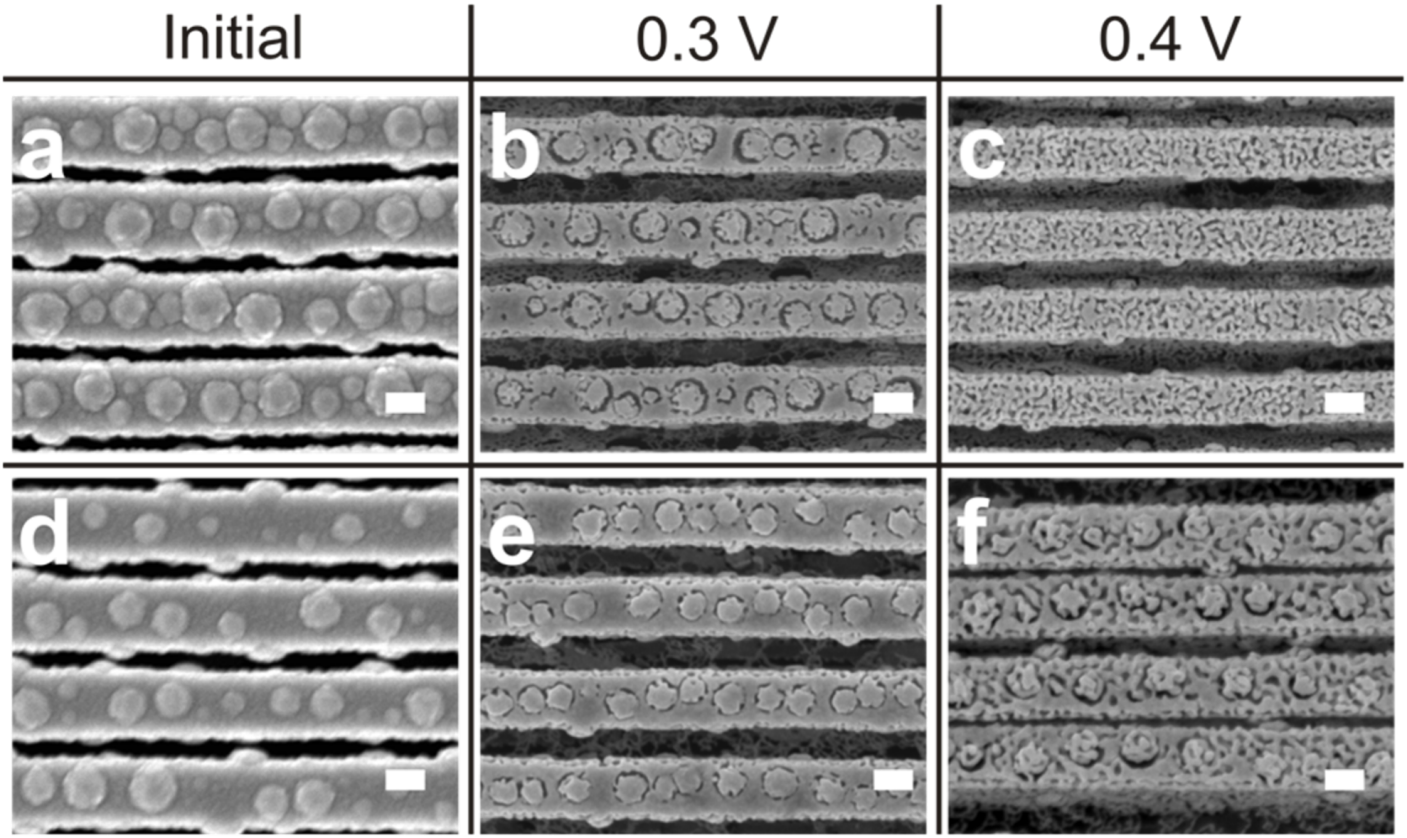
Influence of the dealloying voltage on the formation of gold–copper with a bubbled morphology. SEM image of the Au–Cu nanowires with an initial Au content of 18 atom % (a–c) and 23 atom % (d–f) before (a,d) and after dealloying for 5 min at 0.3 V (b,e) and 0.4 V (c,f). Scale bar: 100 nm.

After optimizing the growth process as described above, we explored the possibility of fabricating porous nanowires with a nodular morphology. For this purpose, we applied an electrochemical dealloying process to Au–Cu alloy nanowires, similarly to the conditions used in our previous work [24], to selectively leach copper from the alloy leaving behind a porous skeleton of gold [25–31]. In Figure 5 are presented plan-view SEM images showing the impact of the dealloying potential on Au–Cu nanowires with a nodular morphology initially containing 18 and 23 atom % gold. These two gold compositions were carefully selected within an experimental window in order to avoid surface passivation or the delamination of the wires during the dealloying process [24]. For this study, a substrate with high amounts of photoresist residues was used. The initial diameter of the nanowires was fixed to 200 nm and the dealloying time was fixed to 5 min. The as-grown nanowires (Figure 5a and 5d) exhibit a nodular morphology with a diameter of about 90 nm, for 18 atom % gold, and about 140 nm, for 23 atom % gold. When dealloying the nanowires with an initial Au content of 18 atom % at a voltage of 0.3 V (Figure 5b), a preferential etching takes place around the hillocks resulting in the formation of ring-shaped pores. Such preferential etching is related to the presence of boundaries between the hillocks and the rest of the nanowire body. They act as a channel promoting the propagation of the electrolyte within the material during the dealloying process. When the electrolyte penetrates through these boundaries, the hillocks become completely surrounded by the electrolyte resulting in an increased dissolution of copper from the alloy. As a consequence, ring-shaped pores appear around the hillocks and the diameter of the latter drops from 150 to 115 nm. When increasing the dealloying voltage to 0.4 V, for 18 atom % of gold (Figure 5c), the hillocks almost disappear and the ring-shaped pores can hardly be distinguished from the rest of the nanowire. Further increasing the dealloying voltage to 0.5 V results in the complete delamination of the nanowires. When nanowires with 23 atom % of initial Au content (Figure 5d) are dealloyed at 0.3 V (Figure 5e), only tiny pores form around the hillocks. The limited porosity is probably related to a surface passivation effect due to the relatively high initial content of Au. Further increasing the dealloying voltage to 0.4 V (Figure 5f) results in the generation of nanoporosity very similar to the one observed in case of Au–Cu nanowires not containing any sort of hillocks [24]. Increasing the dealloying voltage to 0.5 V results in an increase in the porosity within the material (Supporting Information File 1, Figure S4).

## Conclusion

To summarize, we have shown the benefit of using substrates with surface defects to grow metal nanowires with complex surface topography, which afterwards can be engineered on the nanoscale. We investigated the influence of the defects size and density on the final morphology of the nanowires. We demonstrated that the deposition time can impact the size of the hillocks formed over the surface of the nanowires: Increasing the deposition time results in a linear increase in hillock diameter. Furthermore, by applying a dealloying process to such Au–Cu alloy nanowires we have shown that nanowires with ring-shaped pores can be created. The diversity of pore size and shape coupled to the alignment and periodic organization of the nanowires is expected to promote the SERS effect originating from such gold nanostructures.

## Experimental

### Nanopatterned substrates

The nanopatterned silicon substrates used as a template to grow the nanowires were prepared using a two-step approach consisting of laser interference lithography using a NR7-250P photoresist followed by deep reactive ion etching. Such substrates consist of nanograted silicon structures (120 nm in width and 1000 nm in height) of 220 nm in pitch with an aspect ratio of about 6.

### Growth of nanowires

The Cu nanowires were synthesized by DC sputtering in pure argon plasma of a Cu target in a confocal configuration (diameter: 76.2 mm; purity: 99.99%). The distance between the targets and the substrate was 130 mm and the angle between the magnetron source axis and the normal to the substrate was 30°. The Au–Cu alloy nanowires were synthesized by DC co-sputtering in pure argon plasma of an Au (diameter: 76.2 mm; purity: 99.99%) and a Cu (diameter: 76.2 mm; purity: 99.99%) targets in confocal geometry. The electrical power applied to the Cu target was fixed to 300 W whereas the one applied to the Au target fixed to 50 and 70 W yielding nanowires with 18 and 23 atom % of Au, respectively. All depositions were carried out at a pressure of 0.5 Pa without applying any intentional heating to the substrate. For all the depositions, the base pressure was less than 4 × 10^−5^ Pa and the rotation speed of the substrate was fixed to 5 rpm.

### Electrochemical dealloying process

The porous gold nanowires were prepared by electrochemical dealloying of Au–Cu alloy nanowires. These experiments consist in the selective dissolution of Cu by an anodic process. All experiments were performed with a Biologic SP-300 potentiostat. A platinum wire and a saturated calomel electrode (SCE) served as the counter and the reference electrode, respectively. All working electrode potentials are provided with respect to the SCE reference electrode. Electrochemical treatments were performed in diluted H_2_SO_4_ at 0.1 M (pH less than 1) as supporting electrolyte. This condition is selected according to Pourbaix diagram of copper [32]. The contact to the working electrode (i.e., Au–Cu nanowire arrays) was made through a crocodile clip at the tip of a sample injecting the current along the nanowire axes. The treated geometrical surface was typically around 0.5 cm^2^ (the samples were 1 cm in length and 0.5 cm in width). An analysis by cyclic voltametry (between −0.2 V and the dealloying potential at 50 mV·s^−1^) was made before and after the dealloying process to check the current injection in the nanowires and the Cu removal. The dealloying was performed by applying an anodic potential (typically from 0.3 V to 0.5 V) for 5 min. During the dealloying process, magnetic stirring (650 rpm) was applied to ensure a better diffusion of the Cu^2+^ ions leached during the process. After the treatment, the samples were removed from the electrolytic solution, dipped in distilled water and then rinsed with methanol.

### Characterization

The SEM images were recorded using a JEOL JSM 7600 F microscope operating at 5 kV. The AFM images were recorded using a NanoWizard 3 from JPK instruments. TEM imaging was performed on a Hitachi H9000-NAR microscope (LaB_6_ filament, 300 kV, Scherzer resolution: 0.18 nm). For the TEM analyses, the nanowires were sliced with an ultra-microtome to a thickness of around 100 nm and dispersed on a carbon-coated TEM grid.

## Supporting Information

File 1 Additional figures.
