# Supplementary material for: Dealloying of gold–copper alloy nanowires: From hillocks to ring-shaped nanopores
Source: Beilstein J Nanotechnol. 2016 Sep 29;7:1361–7. doi: 10.3762/bjnano.7.127 (PMC5082475; doi:10.3762/bjnano.7.127)
Supplement: File 1 — Additional figures. [file Beilstein_J_Nanotechnol-07-1361-s001.pdf]

Supporting Information

for

Dealloying of gold–copper alloy nanowires: From hillocks to ring-shaped nanopores

Adrien Chauvin[1], Cyril Delacôte[2], Mohammed Boujtita[2], Benoit Angleraud[1], Junjun Ding[3], Chang-Hwan Choi[3], Pierre-Yves Tessier[1], and Abdel-Aziz El Mel*[1]

Address: [1]Institut des Matériaux Jean Rouxel, IMN, Université de Nantes, CNRS, 2 rue de la Houssinière B.P. 32229, 44322 Nantes cedex 3, France, [2]CEISAM, Université de Nantes, CNRS, 2 rue de la Houssinière, 44322 Nantes Cedex 3, France and [3]Department of Mechanical Engineering, Stevens Institute of Technology, Hoboken, NJ 07030, USA

Email: Abdel-Aziz El Mel - Abdelaziz.elmel@cnrs-imn.fr

* Corresponding author

Additional figures

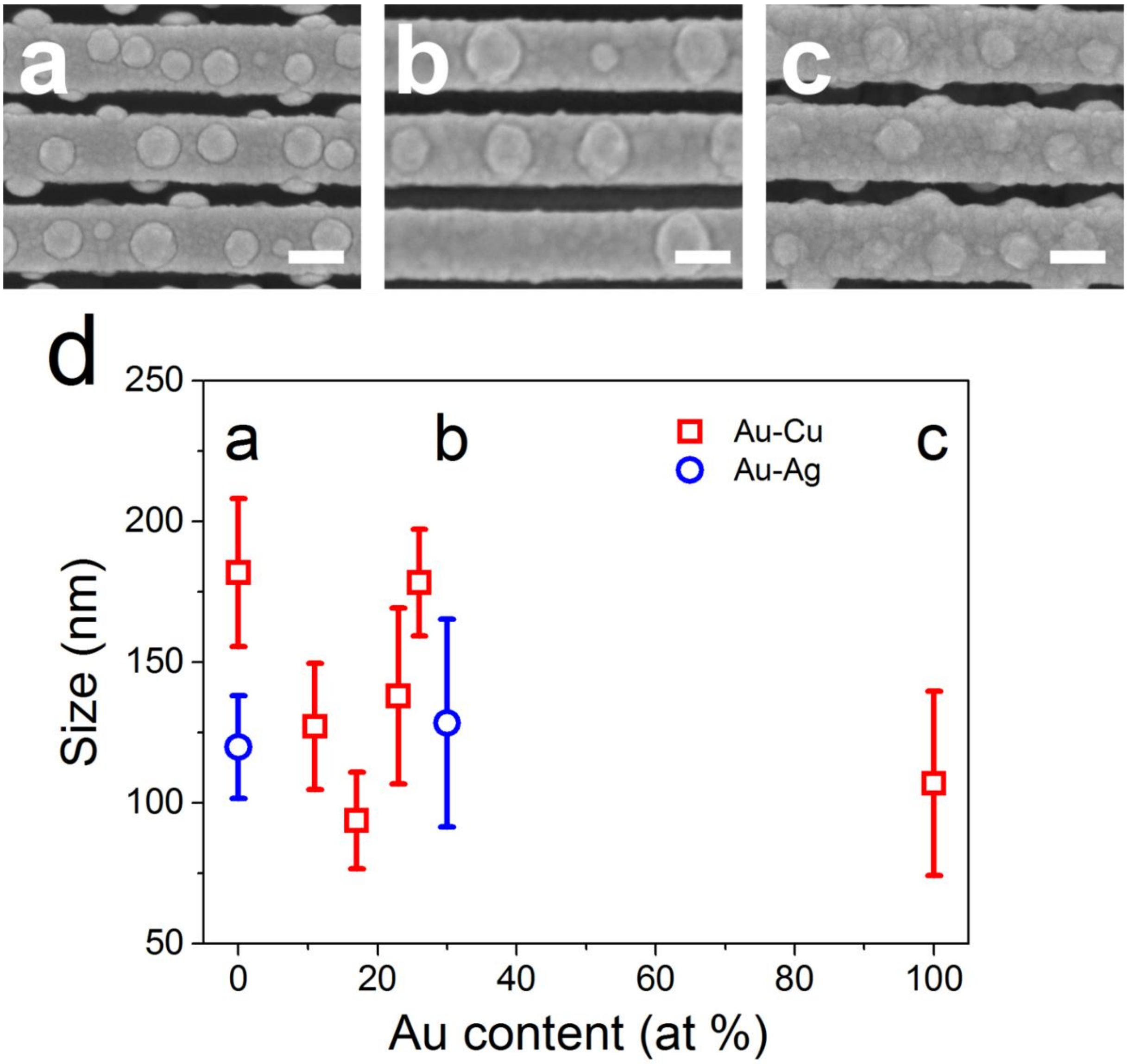

Figure S1: Influence of materials on hillocks diameter for the different metals deposition. Plan view SEM micrograph of (a) pure silver, (b) Ag–Au alloy with 30 at % of silver and (c) pure gold nanowires. Scale bar: 200 nm. (d) Evolution of the hillocks diameters for two different alloys: Au/Cu (open red square) and Au/Ag (open blue circle).

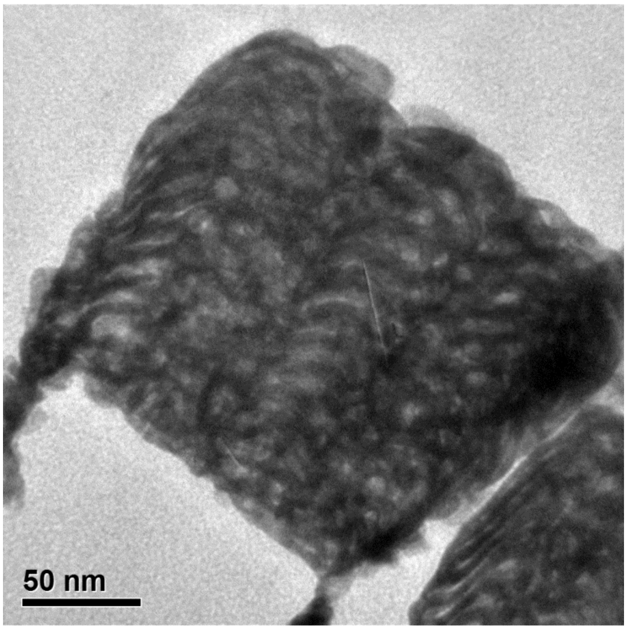

Figure S2: Cross-section TEM image of a Au–Cu nanowire showing the presence of hillocks within the nanowire exhibiting a conical shape.

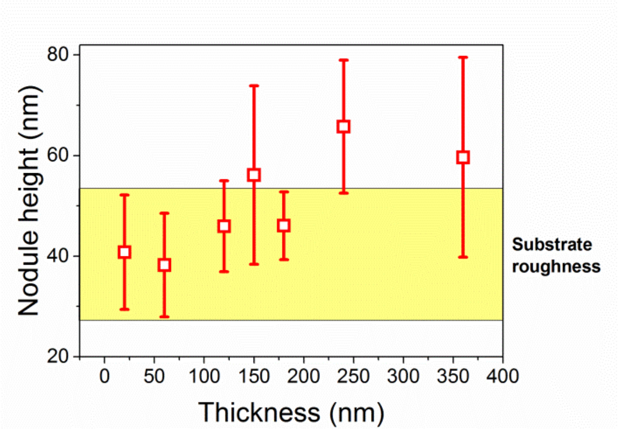

Figure S3: Influence of nodule height after different deposition duration of copper in comparison with the substrate roughness.

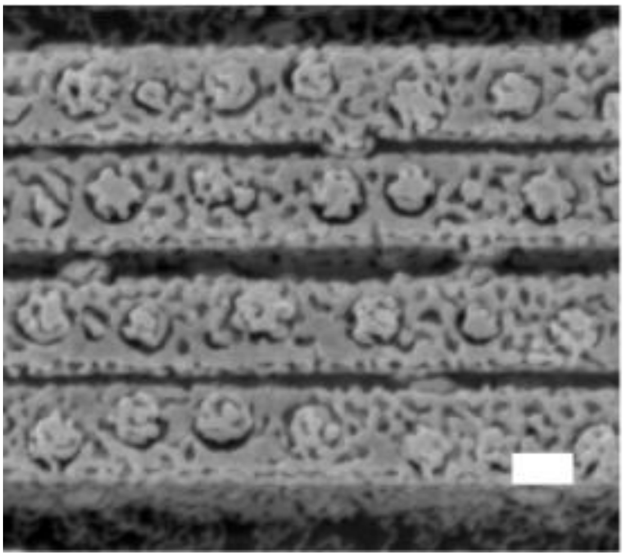

Figure S4: SEM image of Au–Cu nanowires with 23 at % of initial gold content after dealloying for 5 min at 0.5 V. Scale bar: 100 nm.
